# PET Imaging of Atherosclerotic Disease: Advancing Plaque Assessment from Anatomy to Pathophysiology

**DOI:** 10.1007/s11883-016-0584-3

**Published:** 2016-04-23

**Authors:** Nicholas R. Evans, Jason M. Tarkin, Mohammed M. Chowdhury, Elizabeth A. Warburton, James H. F. Rudd

**Affiliations:** Department of Clinical Neurosciences, University of Cambridge, Cambridge Biomedical Campus, Hills Road, Cambridge, CB2 0QQ UK; Division of Cardiovascular Medicine, University of Cambridge, Cambridge Biomedical Campus, Cambridge, CB2 0QQ UK; Division of Vascular and Endovascular Surgery, Cambridge University Hospitals NHS Foundation Trust, Cambridge Biomedical Campus, Cambridge, CB2 0QQ UK

**Keywords:** Atherosclerosis, Positron emission tomography, Coronary artery disease, Carotid stenosis

## Abstract

Atherosclerosis is a leading cause of morbidity and mortality. It is now widely recognized that the disease is more than simply a flow-limiting process and that the atheromatous plaque represents a nidus for inflammation with a consequent risk of plaque rupture and atherothrombosis, leading to myocardial infarction or stroke. However, widely used conventional clinical imaging techniques remain anatomically focused, assessing only the degree of arterial stenosis caused by plaques. Positron emission tomography (PET) has allowed the metabolic processes within the plaque to be detected and quantified directly. The increasing armory of radiotracers has facilitated the imaging of distinct metabolic aspects of atherogenesis and plaque destabilization, including macrophage-mediated inflammatory change, hypoxia, and microcalcification. This imaging modality has not only furthered our understanding of the disease process in vivo with new insights into mechanisms but has also been utilized as a non-invasive endpoint measure in the development of novel treatments for atherosclerotic disease. This review provides grounding in the principles of PET imaging of atherosclerosis, the radioligands in use and in development, its research and clinical applications, and future developments for the field.

## Introduction

Atherosclerosis is a leading cause of morbidity and mortality in the Western world. It is a systemic inflammatory disease that develops over decades, through initial vascular endothelial dysfunction, circulating monocyte recruitment and accumulation, maturation into a necrotic core, atheroma plaque destabilization, and finally plaque rupture [1, 2]. Plaque rupture, and subsequent atherothrombosis, is the primary etiology for myocardial infarction, while large vessel (carotid) atherosclerosis accounts for around one third of ischemic stroke cases [3]. As a reflection of the systemic nature of atherosclerosis, concomitant disease in both coronary and carotid arteries is estimated to occur in 28–58 % of asymptomatic individuals [4–6].

A challenge facing the clinical management of atherosclerosis is differentiating between “stable” and “vulnerable” atherosclerotic plaques, those at risk of rupture and symptomatic atherothrombosis. In clinical practice, the most commonly used carotid imaging modalities are computed tomography (CT) angiography and Doppler ultrasound. In coronary artery disease, while invasive angiography remains the gold standard anatomical imaging technique, non-invasive modalities (CT angiography or perfusion) are increasingly being used for individuals with stable symptoms and low to moderate risk profiles. Within stroke care, medical versus surgical management of carotid atherosclerosis is determined by the degree of luminal stenosis on these investigations. However, this simple anatomical criterion fails to consider other plaque characteristics associated with risk of rupture. By combining samples from the Oxford Plaque Study and the Athero-Express Study, Howard et al. analyzed a pooled sample of 1640 symptomatic plaques. From this pooled data, they showed that plaque thrombus, fibrous content, macrophage infiltration, high microvessel density, and overall plaque instability were each significantly associated with predicted stroke risk [7].

Non-stenotic carotid atheroma has been implicated as a cause of previously classified “cryptogenic” stroke, with non-stenotic plaques demonstrating high-risk morphological features (hemorrhage, thrombus, or fibrous cap rupture) having a higher association with ischemic stroke than those without high-risk features [8, 9]. Furthermore, intravascular imaging of coronary artery atherosclerosis has demonstrated that a significant atheroma burden with a high risk of subsequent cardiac events may be present in the absence of luminal stenosis due to outward artery remodeling [10–12]. Similar findings of high-risk plaques in the presence of non-obstructive lesions have been shown in humans with imaging of coronary remodeling using CT [13, 14].

These limitations of conventional anatomical imaging in the assessment of atherosclerosis have led to increased interest in non-invasive imaging methods to identify features of plaque vulnerability and disease activity. Positron emission tomography (PET) is one such imaging modality that can detect and quantify the pathophysiological processes associated with atherogenesis and subsequent plaque destabilization. PET imaging was originally developed in the mid-twentieth century and is now used routinely in oncological clinical care, though its use as a research tool to measure pathophysiological processes in atherosclerosis is more recent, beginning in 2002. These early atherosclerosis PET imaging studies showed proof of principle for identification of symptomatic carotid atheroma in human subjects, and subsequent animal and human studies provided histological validation. In the intervening 15 years, both animal and human studies have been instrumental in the understanding of the disease process through the development of new PET radiotracers.

## Principles of PET/CT

The complex nature of atherogenesis provides a range of pathophysiological pathways that may be exploited as targets for imaging, many of which are amenable to PET. These targets include inflammation, through hypoxia and apoptosis, to microcalcification. PET utilizes positron-emitting radioligands that accumulate at these different biological processes of interest, their accrual within regions of interest (ROIs) resulting in a localized concentration of emitted positrons that quickly encounter electrons in neighboring tissues, leading to annihilation reactions. Such reactions result in the emission of gamma photons that can be detected by scintillation detectors in the PET scanner. Regions of tracer uptake detected by PET must be co-registered with CT imaging (PET/CT) or magnetic resonance imaging (PET/MRI) to localize the pathophysiological processes to an anatomical location.

A major advantage of PET is its very high sensitivity, allowing picomolar tracer concentrations to be detected that can be used to quantify the biological processes of interest. The most appropriate method for measuring radiotracer activity in vascular tissue remains a subject of debate and is discussed in later sections. The conventional measurement methods are the standardized uptake value (SUV) and tissue to background ratio (TBR). SUV represents the ratio of radiotracer concentration in the target tissue to the injected radiotracer activity adjusted for weight. SUV may be further analyzed as SUV_max_ and SUV_mean_. The SUV_max_ is calculated using the highest tissue radiotracer concentration in the ROI, while SUV_mean_ is calculated using the mean tissue radiotracer concentration within the whole ROI. In contrast, TBR was devised to correct for blood uptake of radiotracer, the “blood pooling” effect. TBR is calculated as the ratio of the SUV of the arterial wall to the SUV in the mid-lumen of a large vein with no evident spill-over effect from neighboring tissues [15].

## PET Radioligands

Specific radioligands can be used in PET imaging to target the metabolic processes involved in atherogenesis and plaque disruption. Broadly, the main pathophysiological processes associated with plaque vulnerability can be split into (i) inflammation (with radioligands targeting macrophages, including ^18^F-fluorodeoxyglucose, somatostatin receptor ligands, and translocator protein ligands), (ii) microcalcification (^18^F-sodium fluoride), and (iii) hypoxia (^18^F-fluoromisonidazole).

While the technique is non-invasive, exposure to both the radiotracer and CT scan involves ionizing radiation. A 250 MBq dose of ^18^F-fluorodeoxyglucose involves a radiation exposure of 5 mSv, in addition to the 0.45 mSv from the CT scan required for attenuation correction. Radiotracers themselves have been shown to have an excellent safety profile, with a review of 81,801 radiopharmaceutical doses showing no recorded adverse reactions [16].

### ^18^F-Fluorodeoxyglucose

**Fig. 1:**
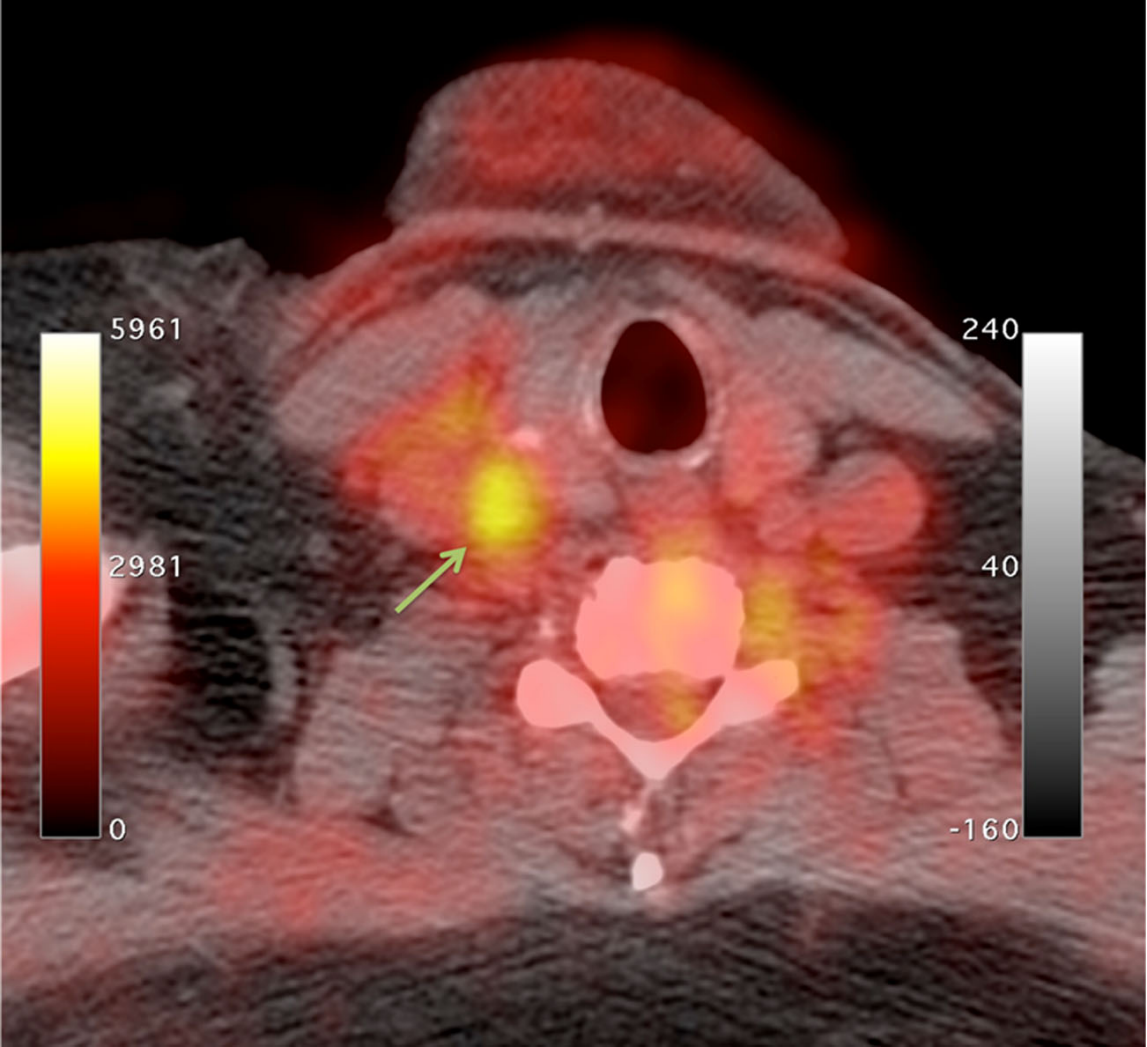
FDG-PET/CT showing high radiotracer uptake in the right common carotid artery (*arrow*)

^18^F-fluorodeoxyglucose (FDG) is the mainstay radioligand in PET imaging and consequently has been the most common radioligand used in imaging studies of atherosclerosis. Originally used for malignancy staging, incidental findings of FDG accumulation in arterial territories during whole-body scans heralded its utility for detecting and quantifying inflammation within atheroma [17]. FDG, a radionucleotide analog of glucose, accumulates intracellularly in proportion to cellular demand for glucose. It is taken up into cells via facilitated glucose transporter member (GLUT) 1 and 3, which are upregulated during atherogenesis due to hypoxia within the atheroma core and once inside the cytoplasm undergoes phosphorylation by hexokinase to become ^18^F-FDG-6-phosphate. ^18^F-FDG-6-phosphate lacks a 2′ hydroxyl group and consequently is unable to enter the Krebs cycle and undergo glycolysis, subsequently diffusing slowly out of the cell. This resulting accumulation is readily quantifiable and can be used as a sensitive measure of metabolic activity, particularly given its very high signal-to-noise ratios in tissues without high metabolic activity (such as normal vessel wall and blood). The high concentration of proinflammatory macrophages in the vulnerable plaque provides such a tissue with a high metabolic activity (Fig. 1).

FDG-PET’s ability to measure plaque inflammation non-invasively in a symptomatic population was demonstrated in early work by Rudd et al. where FDG uptake differentiated between symptomatic and asymptomatic carotid atheroma in human subjects [18], a finding that has been corroborated in recent larger studies [19•]. This increased uptake of FDG detected by PET/CT has been shown to correlate with histological macrophage density in animal models [20, 21] and excised atheroma following carotid endarterectomy [15, 22].

FDG uptake has been shown to identify symptomatic carotid plaques that were non-stenotic on high-resolution MRI, supporting the concept that the severity of stenosis is not the sole determinant for symptomatic plaque rupture [23]. However, this small study contrasted with another small pilot study that showed that although FDG uptake was higher in symptomatic arteries, uptake also correlated with the degree of stenosis [24]. In a large FDG-PET study by Tahara et al., only 29 % of asymptomatic individuals with carotid atherosclerosis found on Doppler screening had FDG uptake within the plaque, with no difference observed in the carotid intima-media thickness between inflamed and non-inflamed plaques [25]. The observed association between increased FDG uptake with high-risk morphological plaque features measured by CT reinforces this finding and the shortcomings of solely anatomical assessments of stenosis [26]. Multimodal imaging studies using FDG-PET and MRI have allowed comparison of tracer uptake with more accurate assessment of plaque morphological features. Silvera et al. imaged individuals with vascular risk factors and found FDG TBR_mean_ to be higher for lipid-rich plaques, which are often vulnerable to rupture, than for collagen-rich or calcified plaques with a lower risk of rupture [27].

In addition to its relation with the index plaque rupture, higher FDG uptake in carotid atheroma has been shown to be associated with a higher risk of recurrent cerebrovascular events, independent of the degree of luminal stenosis [28]. This is supported by an association of higher FDG uptake and microemboli detected by transcranial Doppler [29•].

**Fig. 2:**
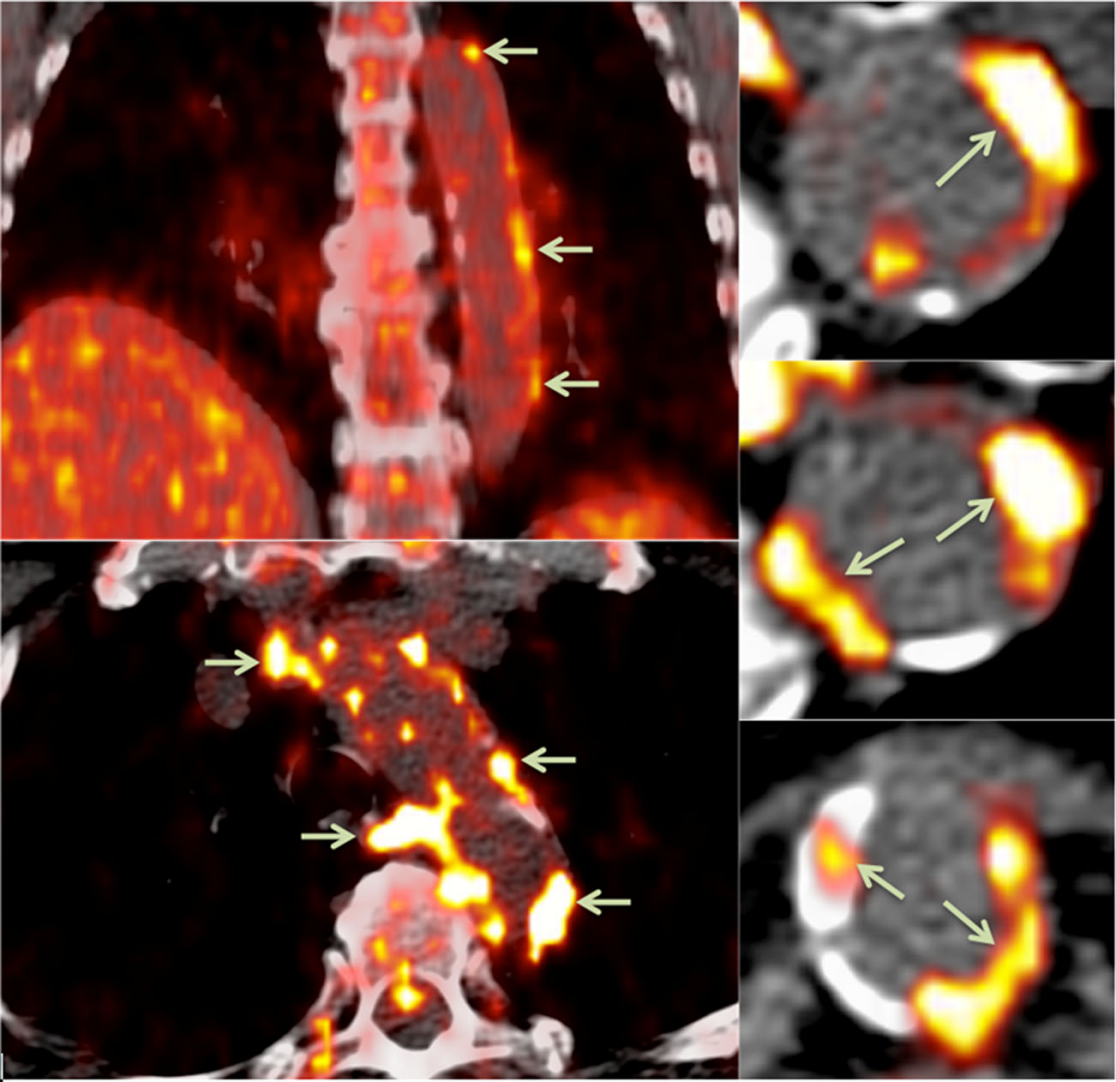
FDG-PET/CT showing areas of focal radiotracer uptake in the wall of the descending aorta (*arrows*)

FDG-PET techniques have helped elucidate the systemic nature of atherosclerosis (Fig. 2). FDG uptake correlates closely between neighboring arterial territories, suggesting a global upregulation of inflammation rather than a localized phenomenon [30••]. Joshi et al. demonstrated that FDG uptake in the aorta reflected the clinical severity of coronary syndromes, with a 20 % higher TBR in the aortas of those with a recent myocardial infarction than those with stable angina. Furthermore, within the group with myocardial infarcts, the aortic FDG uptake was higher for those with an ST elevation myocardial infarction than those with a non-ST elevation myocardial infarction [31]. Similarly, carotid SUV_mean_ and TBR_mean_ are significantly higher for cohorts with acute coronary syndrome than for those with chronic stable angina [32].

A possible mechanism for this relationship has been demonstrated through the association between focal arterial inflammation and systemic metabolic syndrome. Carotid TBR_max_ is higher in both non-obese individuals with metabolic syndrome and obese individuals without metabolic syndrome compared to non-obese individuals without metabolic syndrome [33]. Furthermore, both low-density lipoprotein and total cholesterol have been shown to be independently associated with FDG uptake [34, 35]. These findings go some way to explaining the association between higher Framingham risk factor scores and higher TBR.

The interface between systemic inflammation driving atheroma inflammation has been suggested by the association between periodontal inflammation and inflammatory activity within atheroma, both of which reduced in response to atorvastatin and were strongly correlated [36, 37]. Serum inflammatory markers have been found to be associated with an increased risk of cardiovascular events, potentially representing either a cause or result of an upregulated inflammatory response [38–40]. Myeloperoxidase levels are associated with a higher FDG TBR in carotid diseased segments, independent of other conventional cardiovascular risk factors, although no independent relationship was found for high-sensitivity C-reactive protein (hsCRP), interleukin 6 (IL-6), or matrix-metalloproteinase 9 (MMP-9) [41]. However, other studies have provided conflicting results, with an association between higher TBR and higher levels of hsCRP [33, 42]. In comparison to blood biomarkers, FDG uptake can localize to focal sites of high inflammatory activity. The focal plaque inflammation on a background of a systemic inflammatory reaction may account for the finding of neighboring regions of FDG uptake.

Recent FDG-PET studies have continued to provide insights into the interactions and contributions of different aspects of the inflammatory process within atherosclerosis. In a prospective FDG-PET study, regions of the aorta with high SUV were more likely to develop calcification on subsequent CT imaging, independent of cardiovascular risk factors [43]. Furthermore, in a substudy of the dal-PLAQUE study, Joshi et al. found that FDG TBRs reduced over 6 months if carotid calcification was absent, though there was no interval change in tracer uptake in carotid arteries where calcification was present [44]. Consequently, the authors concluded that calcium deposition is a propagating factor for ongoing arterial inflammation.

The accumulation of cholesterol crystals within atheroma has also been shown to promote plaque inflammation and rupture in animal models, while crystal content found in human carotid histology was found to be strongly associated with plaque disruption, thrombus, and symptoms [45–47]. Though there is no PET radioligand for imaging cholesterol crystals, instead relying on electron microscopy, their effect on inflammation within the plaque may be measured. Patel et al. showed that ezetimibe reduced the cholesterol crystal density on electron microscopy in the aortas of atherosclerotic rabbits, with a corresponding decrease in inflammation as quantified by FDG uptake (SUV_max_), CRP, and MMP-9 levels [48].

FDG-PET has a number of advantages, but also limitations, compared to other imaging modalities. It is highly reproducible with high intra-observer and inter-observer agreement [49]. PET/CT using FDG has a high sensitivity for detecting inflammation in plaques, but its utility to detect inflammation may be hindered when the ROI is in close proximity to other tissues with tracer uptake due to high resting metabolic rates (such as neurons and myocardial tissue) and may be further compounded by the low spatial resolution of PET (approximately 3 mm). Solutions for both of these weaknesses are currently being developed with the advent of more cell-specific ligands and PET/MRI. Dynamic contrast enhanced (DCE) MRI has been shown to have superior spatial resolution than PET/CT, and contrast enhancement has been particularly effective in the assessment of fibrous cap thickness and lipid core volume, where the former enhances while the latter fails to enhance [50]. Multicontrast-weighted MRI has been used in surveillance of asymptomatic plaques and demonstrated larger lipid cores, thicker MWT, thin/ruptured fibrous caps, and intraplaque hemorrhage to each have significantly increased hazard ratios for subsequent symptomatic events [51]. However, while MRI offers an effective method for imaging morphological features associated with plaque vulnerability, it remains dependent on accurate coil placement as well as the reproducibility of technical sequences and image generation. These considerations, along with a desire to image directly the biological activity in the plaque, has led to DCE MRI and PET/CT providing complementary imaging of the plaque, though the advent of PET/MRI may enable a fusion of these techniques and is discussed further below.

### Translocator Protein Ligands

While FDG-PET has become a mainstay of metabolic imaging in atherosclerosis in a relatively short time, its lack of specificity means that proximity of the artery of interest to other highly metabolically active structures, such as the myocardium, limits its utility. Alternative radiotracers targeting macrophage-driven inflammation via their expression of translocator protein (TSPO) have been investigated with the goal of providing higher specificity than that offered by FDG. ^11^C-PK11195 (^11^C-*N*-methyl-*N*-[1-methylpropyl]-1-[2-chlorophenyl]-isoquinoline-3-carboxamide) targets TSPO expressed on macrophages and microglia and has been shown to detect these inflammatory cells in atheroma and around the stroke penumbra, respectively. Specific to atherosclerosis, ^11^C-PK11195 uptake was found to be higher in inflamed than non-inflamed plaques in a mouse model, though its utility as a tracer was limited due to a non-significant difference between plaque and healthy vessel wall [52]. This likely reflects the ubiquitous nature of TSPO expression by a range of cells and organs, despite the upregulation in activated plaque macrophages, but may also reflect the limitations of using mouse models of plaque, especially vulnerable plaques, compared to humans. Subsequent studies in human subjects have shown more promise, with ^11^C-PK11195 TBR found to be higher in symptomatic versus asymptomatic carotid arteries (TBR 1.06 ± 0.2 and 0.86 ± 0.11, respectively, *p* = 0.001) despite a lower grade of stenosis in asymptomatic arteries [53]. ^11^C-PK11195 uptake has been found to co-localize with activated macrophages using autoradiography and CD68 staining of ex vivo carotid histology [54, 55]. However, the ubiquitous uptake in healthy vessel wall may limit the utility of ^11^C-PK11195-PET in clinical atherosclerosis imaging.

Newer TSPO ligands are in development, labeled with ^18^F rather than ^11^C. There has been increasing interest in ^18^F-GE-180 (*S-N*,*N-*diethyl-9-[2-^18^F-fluoroethyl]-5-methoxy 2,3,4,9-tetrahydro-1H-carbamazole-4-carboxamide) to replace PK-11195 as the TPSO ligand of choice to image neuroinflammation and potentially atherosclerotic plaque inflammation. Animal models have shown ^18^F-GE-180 to have a significantly higher binding potential than that of PK-11195, improved signal-to-noise ratio, and lower non-specific binding in and around infarcted cerebral tissue [56, 57]. The use of ^18^F in contrast to ^11^C has other benefits including a longer half-life. However, the utility of such second-generation TSPO radioligands in PET imaging may be limited owing to variable receptor binding affinity due to genetic polymorphisms [58–60]. Further work is required to assess the utility of ^18^F-GE-180 and other second-generation TSPO ligands for imaging both plaque inflammation and neuroinflammation.

### ^68^Ga-DOTATATE and ^64^Cu-DOTATATE

Another clinically available PET radioligand under investigation for use in atherosclerosis imaging is DOTATATE ([1,4,7,10-tetraazacyclododecane-*N*,*N′*,*N″*,*N″′*-tetraacetic acid]-*d*-Phe1,Tyr3-octrotate). DOTATATE binds to somatostatin receptor subtype-2 (SST_2_), which appears to be upregulated on the surface of activated macrophages [61, 62]. The low physiological expression of SST_2_ by the myocardium suggests that this tracer may be advantageous for targeting disease in the coronary arteries.

Vascular ^68^Ga-DOTATATE uptake has been imaged in asymptomatic individuals with cardiovascular risk factors and coronary calcification [63–65] and in aortic atherosclerotic plaques in a mouse model [66]. In a retrospective series of DOTATATE-PET imaging performed in oncological practice, Rominger et al. found ^68^Ga-DOTATATE-PET to have an excellent intra-reader and inter-reader reproducibility for TBR readings in the left anterior descending coronary artery (intra-class correlation coefficients of 0.97 and 0.94, respectively) [65].

^64^Cu-DOTATATE has also been investigated for use in carotid imaging. The longer half-life of ^64^Cu compared to ^68^Ga (12.7 h versus 68 min) and shorter maximum positron range provide several theoretical advantages, although this must be balanced against the wider availability of the generator-produced ^68^Ga compared to the cyclotron-produced ^64^Cu. In a proof-of-principle study using PET/MRI, Pedersen et al. demonstrated carotid ^64^Cu-DOTATATE uptake correlated with gene expression of macrophage markers CD68 and CD163 using univariable analysis, though only correlation with CD163 expression remained significant on multivariable analysis [67]. DOTATATE’s propensity toward CD163+ macrophages, as well as findings of atheromatous regions with DOTATATE but no FDG uptake, suggests that DOTATATE is able to identify a different component of the inflammatory process compared to conventional FDG-PET [65, 67]. Increased ^64^Cu-DOTATATE signal has also been reported in individuals with cardiovascular risk factors in a retrospective study [68].

Although DOTATATE-PET has shown early promise as a potential candidate radiotracer for imaging inflammation in atherosclerosis, this newer technique has not yet been tested in large human studies in individuals with symptomatic disease. Further prospective clinical studies and histological validation are needed.

### ^18^F-Sodium Fluoride

Inflammation is not the sole metabolic process contributing to plaque vulnerability. Inflammation within the atheroma can promote microcalcification, the formation of deposits of calcium smaller than 50 μm, through cytokine-mediated promotion of osteoblast-like cells derived from vascular smooth muscle cells [69–71]. Plaque macrophage burden showed a strong association with osteoblastic activity in the aortas of hyperlipidemic mice, and both macrophage burden and osteogenic activity increased with plaque progression [72]. Microcalcification may predispose to plaque rupture either through mechanical disruption to the fibrous cap and/or provoking ongoing inflammation around the deposit.

**Fig. 3:**
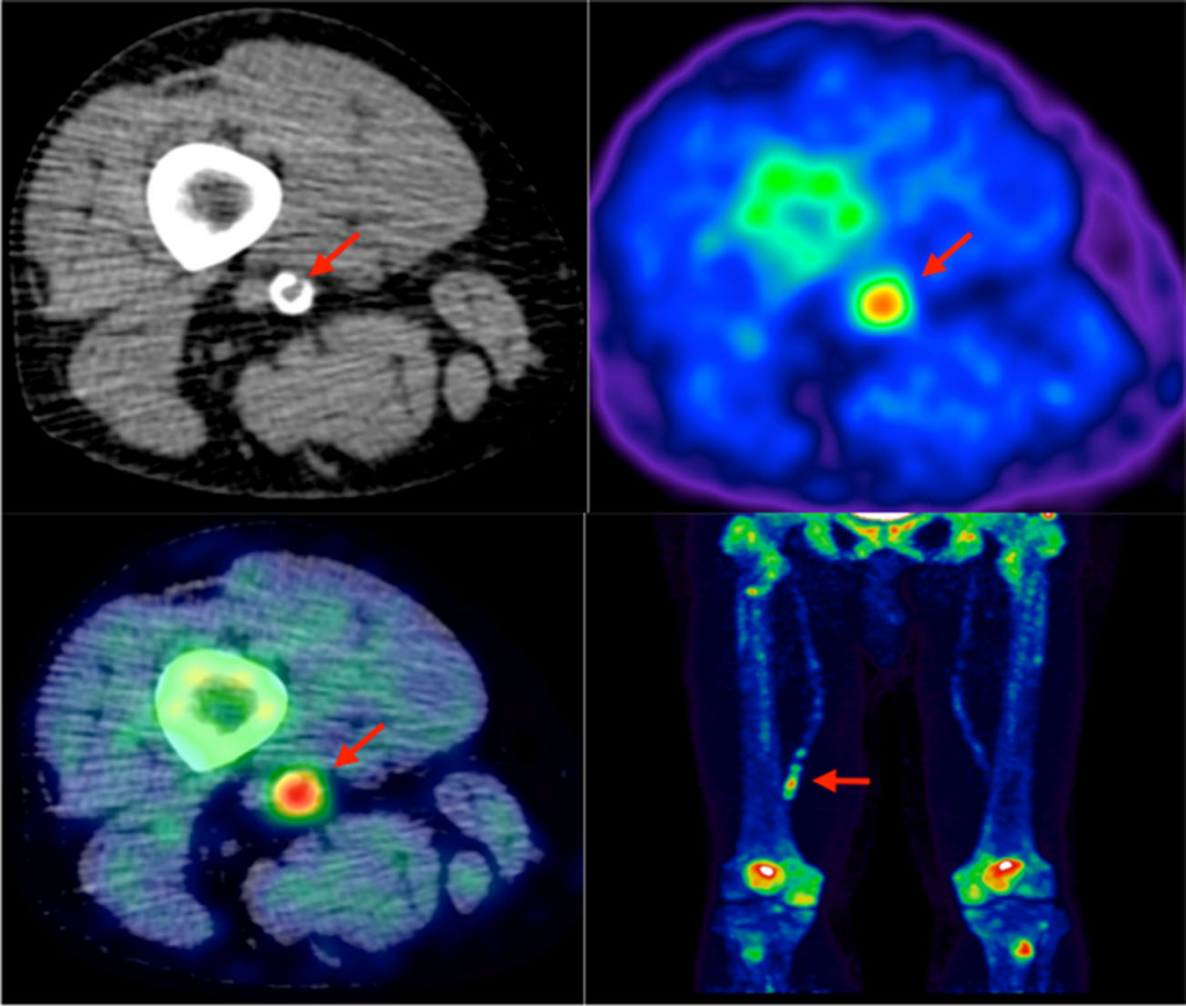
Lower limb ^18^F-NaF imaging: non-contrast CT (*top left*) with a rim of calcification of the vessel, ^18^F-NaF PET (*top right*), and fused ^18^F-NaF PET/CT (*bottom left*) of the superficial femoral artery (*arrow*) at the level of the adductor canal, demonstrating significant vessel uptake in this symptomatic patient. In addition, there is prominent uptake seen in the vessel at the same level on the coronal image (*bottom right*)

^18^F-sodium fluoride (NaF) used in PET imaging is able to identify areas of microcalcification in vivo because the radiolabeled fluoride is taken up at sites of mineralization, where it replaces the hydroxyl group of hydroxylapatite [73] (Fig. 3). Work by Irkle et al. validated clinical NaF-PET using NaF against electron microscopy, autoradiography, and microPET for detecting microcalcification in symptomatic carotid endarterectomy histological specimens. Fluoride was shown to co-localize closely and preferentially bind to pathological mineralization, and the increased surface area of microcalcification relative to macrocalcification resulted in increased tracer uptake [74••].

The feasibility of NaF-PET to identify microcalcification in atherosclerotic plaques was demonstrated by Derlin et al. in a cohort of patients undergoing full-body NaF-PET/CT for oncological staging of bone metastases. This study showed that NaF uptake may occur at sites of macrocalcification, but distinct areas of NaF uptake and macrocalcification occurring in isolation demonstrated that NaF uptake reflects the active mineralization process in microcalcification rather than simply the burden of macrocalcification [75]. Further lack of co-localization between regional macrocalcification and regional NaF uptake has been found in other asymptomatic cohorts, with an inverse relationship between NaF uptake and plaque calcium score [76, 77]. Morbelli et al. demonstrate that the presence of cardiovascular risk factors correlate with NaF uptake but not arterial macrocalcification, though the study’s measurement of uptake across the whole vessel may miss focal concomitant areas of macrocalcification and NaF uptake [76]. Further studies have found that carotid NaF uptake correlated with the presence of cardiovascular risk factors in an asymptomatic population [78]. Derlin et al. performed a dual-tracer PET/CT study using FDG-PET and NaF-PET in a further asymptomatic oncological patient cohort and found that of 215 arterial lesions identified by either tracer, only in 14 (6.5 %) was there concomitant FDG and NaF uptake, implying that macrophage-driven inflammation and microcalcification are two related but distinct processes [79].

A prospective study has shown that NaF TBR_max_ is higher in individuals with coronary artery disease, stable angina, or previous cardiovascular events [80]. Increased tracer uptake has been shown to be associated with symptomatic coronary plaques, with the increased uptake seen in morphologically high risk but unruptured plaques suggesting that uptake reflects the microcalcification process rather than increased surface area following plaque rupture [81••]. In contrast, no significant difference in NaF uptake between symptomatic and asymptomatic carotids was found in a cohort of stroke patients, though the number of patients was small with long bolus to scan times [82].

The inter-rater repeatability of NaF-PET/CT is high, with an intra-class coefficient of 0.99 found in one study [80]. Further improvements in the quantification of microcalcification in coronary atherosclerosis is likely following developments in motion correction resulting in an improved signal-to-noise ratio, reducing signal “spill-over” from motion causing the radiotracer signal (detected over minutes) to fall outside the ROI determined from anatomical scans (taken over seconds) [83].

### Quantifying Hypoxia

Atherosclerosis is often associated with hypoxia, presumably due to an increasing oxygen demand from foam cells. This likely results from reduced diffusion efficiency from lumen to wall as plaque thickness increases [84, 85]. Hypoxia potentiates the inflammatory response via hypoxia-inducible factor-1α (HIF-1α) expressed by macrophages in the core, a response that may be further upregulated by the presence of oxidized low-density lipoprotein [86, 87]. Whereas structural imaging techniques can assess the size of the necrotic core of the plaque, PET imaging using ^18^F-fluoromisonidazole (FMISO) can measure the effects of hypoxia within the core directly. In a hypoxic environment, the FMISO is reduced and, in the absence of reoxidation, remains bound intracellularly. Mateo et al. demonstrated that in a hyperlipidemic rabbit model, FMISO uptake was significantly higher in regions of atheroma than in normal tissue. Furthermore, immunohistochemistry showed the hypoxia to be deep in the core rather than at the luminal boundary [88]. In an early clinical study, FMISO TBR was found to be higher in symptomatic than asymptomatic carotid atherosclerosis, with FMISO uptake correlating with FDG uptake, suggesting that hypoxia is a contributing factor in FDG uptake [89].

Recently, another radioligand targeting plaque hypoxia, ^18^F-HX4 (^18^F-2-(4-((2-nitro-1H-imidazol-1-yl)methyl)-1H-1,2,3-triazol-1-yl)propan-1-ol), has been shown to have specific uptake in regions of plaque with a strong correlation between TBR_max_ and carotid arterial wall dimensions on 3.0-T MRI [90].

## Methodological Considerations

There are a number of methodological and technical considerations in PET/CT assessment of the plaque. At a single study level, these primarily include partial volume correction and methods of tracer uptake quantification. In contrast, the use of different study protocols within the field also has implications for reproducibility and the ability to pool results in larger meta-analyses.

### Partial Volume Correction

Measurement of tracer uptake within the atheroma is dependent upon the size of the atheroma and the resolution of the scanner. The partial-volume effect occurs when limited resolution results in difficulty differentiating the tracer activity of the ROI from the tracer activity of the surrounding tissues. This may lead to “spill-out,” where the tracer signal from the atheroma falls outside of the ROI, and “spill-in” where tracer signal from adjacent tissue falls within the ROI. The combination of these two effects results in partial volume error (PVE). Small atheromatous lesions falling below the spatial resolution of the scanner are particularly at risk of PVE. It is estimated that the dimensions of a homogenous ROI need to be two to three times the spatial resolution of the scanner in order to minimize PVE [91]. Reduction of PVE through partial volume correction (PVC) can be performed using a geometric transfer matrix (GTM), whereby co-registration with a higher resolution modality allows restriction of the tracer signal to a corresponding voxel-based volume of interest that can then undergo further voxel-based adjustment for PVE using an algorithm proposed by Rousset el al. [92]. This method has been applied to atheroma imaging (using co-registration with MRI) and was found to improve quantification of tracer activity and to be highly reproducible [93].

### SUV Versus TBR

Studies conducted to date have varied in their use of SUV or TBR, and the most appropriate quantification method remains a subject of debate [94]. In plaque simulations based on real patient data, marked bias was found in both measured SUV_max_ and SUV_mean_ compared to the modeled values, largely due to the spatial resolution of the reconstructed image typically being more than three times the thickness of the atherosclerotic plaque, resulting in a reduced ability to correct for PVE. Bias was more marked when fewer iterations were used during image reconstruction [94].

In a study of 32 patients undergoing endarterectomy, Niccoli et al. compared the ability of SUV_max_, SUV_mean_, TBR_max_, and TBR_mean_ to differentiate in vivo plaques classified as either inflamed or non-inflamed following histological examination ex vivo. Within the symptomatic arteries, the authors found that the differences for SUV_max_ and SUV_mean_ between inflamed and non-inflamed plaques were non-significant, while both TBR_max_ and TBR_mean_ were able to differentiate between plaques found to be inflamed or non-inflamed on histology [95].

TBR has limitations, and its application is not without its criticisms. Blood-pool SUV has been shown to decrease with increasing injection to scan intervals (mean SUV within the jugular vein was 1.04 ± 0.16 at 1 h, 0.79 ± 0.03 at 2 h, 0.66 ± 0.04 at 3 h). Consequently, plaque TBR values differed significantly between cohorts scanned at 2 and 3 h. In contrast, tissue SUV_max_ did not differ between these time points [96]. Furthermore, impaired renal function will also contribute to increased blood-pool activity. Blood-pool SUV has been found to be inversely proportional to the estimated glomerular filtration rate, resulting in a lower TBR with lower renal clearance [97].

There has been an overall shift toward TBR in arterial PET imaging, but the possible confounders described above must be considered when comparing results both within the same study and against other studies. Standardization of scan methodologies and patient cohorts will help address this.

### Reproducibility

Increasingly, specific radioligands have resulted in improved signal-to-background ratios and reproducibility, with high intra-rater and inter-rater reproducibility reported for NaF and ^68^Ga-DOTATATE [65, 80]. However, a major consideration within the field of vascular PET imaging is the wider reproducibility of individual studies. Typically, most vascular PET studies of symptomatic patients are small, with fewer than 50 participants, due to a combination of sufficient statistical power with small participant numbers balanced against radiation exposure and high economic costs. While the multitude of FDG studies would seem prime for meta-analysis, the heterogeneous patient populations and variations in methodology pose barriers to such analyses. Variations in tracer doses, interval between symptoms and imaging, blood glucose thresholds, and measurement techniques (SUV versus TBR) are a few of the differences in studies that make direct comparison difficult. The effect of the symptom to scan interval on the potential variability of the PET signal is difficult to quantify as radiation exposure is a barrier to longitudinal studies and should therefore be analyzed in any multivariate analysis. Consequently, there is a move toward establishing a recognized common standard for arterial PET imaging. A recent position paper from the European Association of Nuclear Medicine has recommended such common standards for FDG-PET, particularly with regards to injected dose, circulation uptake time, prescan fasting glucose limits, and suggested quantification using TBR in most cases [98]. Adopting a unified approach to scanning protocols and quantification will allow the high inter-rater reproducibility to be exploited, existing data to be pooled into larger meta-analyses, and standardization of multicenter PET imaging studies.

## PET/CT Applications in Atherosclerosis Drug Trials

As well as its utility for understanding the pathophysiology of atherosclerosis, PET/CT has an important application for measuring the effects of drug treatment. FDG-PET has also offered key insights into mechanisms of atheroma stabilization with statins, in particular their observed anti-inflammatory effects upon the atheroma in addition to their effect on lipid profiles [99–101].

The capacity to measure atherosclerotic metabolic processes non-invasively in vivo has been shown to provide a useful endpoint for drug discovery and efficacy trials. The dal-PLAQUE phase 2b randomized clinical trial of dalcetrapib (a modulator of cholesteryl ester transfer protein that raises high-density lipoprotein cholesterol) used FDG uptake as its primary endpoint and demonstrated that there was no safety concerns over the 6 months while on dalcetrapib. In this study, dalcetrapib failed to reduce carotid FDG uptake when compared to placebo, which was consistent with the later randomized placebo-controlled clinical outcome study [102, 103]. FDG-PET endpoints have also been used by Emami et al. who used it to compare the therapeutic effects of BMS-582949 (a p38 mitogen-activated protein kinase inhibitor) against placebo, but again, no significant difference was seen between these two cohorts [104]. This study did reinforce the finding that statin treatment leads to a reduction in FDG uptake in the control group, and this may contribute to the lack of significance between cohorts. These studies serve as an important proof of principle for the use of PET endpoints in randomized clinical trials.

## Future Directions

Recent advances have led to the feasibility of MRI for anatomical co-registration with PET. PET/MRI has a number of potential advantages over PET/CT. MRI, using either contrast enhancement or black-blood imaging, has proven to be an effective non-invasive imaging modality for assessing and quantifying plaque morphological features. The complementary nature of MRI and PET for assessing plaque morphology and metabolism, respectively, is advantageous in the identification of vulnerable plaques [27, 105, 106]. However, currently, this requires two different scans with consequent difficulties for co-registration. Recent small studies on hybrid PET/MRI scanners have shown promising proof-of-principle data for combined morphological and metabolic imaging of atherosclerosis. In an asymptomatic cohort, Ripa et al. found a moderate to good correlation for PET/CT and PET/MRI FDG SUV_max_ (*r* = 0.6) and SUV_mean_ (*r* = 0.8) on carotid vessel-by-vessel comparison, though there was noted a small but significant under-reading for PET/MRI of less than −0.2 for both SUV_max_ and SUV_mean_ [107]. Hyafil et al. performed FDG-PET/MRI in a cohort of 18 cryptogenic strokes for individuals with “non-stenosing” carotid disease (i.e., stenosis measured as ≤50 %) and found that there was a significantly higher proportion of plaques with the highest morphological features of vulnerability (AHA lesion type 6, so-called complicated plaques) in the ipsilateral carotid compared to the side contralateral to the infarct. The FDG TBR_mean_ of both the ipsilateral and contralateral carotid arteries of individuals found to have AHA type 6 lesions was higher than the TBR_mean_ of individuals without complicated carotid disease [108]. Additional larger studies are required to evaluate the utility of PET/MRI, in particular regarding considerations such as attenuation correction and the implications for PVE.

## Conclusions

The evolution of atherosclerosis imaging from anatomical to metabolic imaging has provided novel insights into the pathophysiology underlying atherogenesis and plaque vulnerability. Broad adoption within routine clinical care is currently limited by availability, cost, and radiation exposure. However, as a research tool, it has facilitated the introduction of metabolic endpoints in studies of new treatments for atherosclerosis, as well as a means to measure the therapeutic effects of existing treatments. Translation of PET/CT techniques such as these to the clinical setting will require phase 3 trials where treatment decisions are made on the basis of metabolic imaging data rather than conventional structural imaging. The potential for newer, more specific radioligands and the increasing availability of PET/MRI is likely to advance our understanding of atherosclerosis and help the development of novel therapeutics to combat this important disease.
